# Bioactivity-guided isolation of anti-proliferative compounds from endemic *Centaurea kilaea*

**DOI:** 10.1080/13880209.2016.1255980

**Published:** 2016-12-09

**Authors:** Ali Sen, Suna Ozbas Turan, Leyla Bitis

**Affiliations:** a Department of Pharmacognosy, Faculty of Pharmacy, Marmara University, Istanbul, Turkey;; b Department of Pharmaceutical Biotechnology, Faculty of Pharmacy, Marmara University, Istanbul, Turkey

**Keywords:** Asteraceae, breast cancer, cnicin, cirsimaritin, prostate cancer

## Abstract

**Context:** The genus *Centaurea* L. (Asteraceae) is one of the largest genera in Turkey. Compounds and extracts obtained from different *Centaurea* species have significant anti-cancer activity against various cancer cell lines.

**Objective:** To determine the anti-proliferative activity of isolates from the chloroform extract of *C. kilaea* Boiss.

**Materials and methods:** Eleven compounds were isolated using column chromatography and preparative TLC from the chloroform extract of aerial parts of endemic *C. kilaea*. The structures of the isolated compounds were elucidated by various spectroscopic methods, including UV, ^l^H-NMR and ^13^C-NMR. Anti-proliferative activity of compounds (0.5–50 μg/mL) were measured against one normal cell line (L-929, mouse fibroblast) and three human cancer cell lines (Hela, cervix carcinoma; MCF-7, breast carcinoma; PC-3, prostate carcinoma) using MTT assay. Results were expressed as IC_50_ values.

**Results:** None of the 11 compounds displayed activity against L-929 and HeLa. Two of these compounds, cnicin and cirsimaritin, showed fairly strong activity against MCF-7 and PC-3 with IC_50_ values of 3.25 and 4.3 μg/mL, respectively.

**Discussion and conclusion:** This is the first report on cirsimaritin. Cirsimaritin and cnicin could serve as potential anti-cancer drug candidates against breast and prostate cancer, respectively.

## Introduction

Cancer is a complicated genetic disease defined as uncontrolled growth, invasion, angiogenesis and metastasis, and one of the main causes of death in the world (Moura et al. 2016). According to the American Cancer Society (ACS), deaths resulting from cancer comprise 2–3% of yearly deaths worldwide (Unnati et al. 2013). In Turkey, the age-standardized rate of cancer among males in 2013 was 267.9 per 100,000; for female, the rate is 186.5 per 100,000. Total cancer incidence is 227.2 per 100,000, and also 174,000 new cancer cases in Turkey have been diagnosed in 2013 (TPHI 2016).

The present treatment of cancer is performed by means of chemotherapy, radiation therapy, surgical resection (especially for cancer types such as breast and stomach cancer) or combination of these therapies (Wang et al. 2013). Among these, chemotherapy is the most common cancer treatment, however due to its side effects, it needs to be improved (Ummavathy et al. 2015; Zheng et al. 2016). Complementary and alternative medicine is the most noticeable approach in cancer management. Natural products are valuable sources for new therapeutic compounds. Most of the anti-tumour drugs are generally developed from efficient herbal phytochemicals. Therefore, medicinal plants could be a good source of anti-tumour agents. Herbal-based drugs might be developed after systematic assessment and chemical modification (Zheng et al. 2016).

*Centaurea kilaea* Boiss. (Asteraceae) is one of the 205 taxon of the genus *Centaurea* growing wild in Turkey (Davis 1975; Davis et al. 1988; Güner et al. 2000). Bensouici et al. (2012) reported that *Centaurea* species are rich in flavonoids and sesquiterpene lactones. In traditional medicine, *Centaurea* species are used in the treatment of fever, menstrual disorders, vaginal candidiasis, and liver, kidney, ulcer diseases, and also as an anti-diarrhoeal, stomachic, tonic, appetitive, anti-diabetic, anti-pyretic, diuretic and expectorant (Baytop 1999; Tuzlacı et al. 2010).

Anti-proliferative activity studies conducted on some *Centaurea* species in recent years have found that compounds and various extracts of these species have significant anti-tumour activity (Csupor-Löffler et al. 2009; Rajabi et al. 2009; Csapi et al. 2010; Baykan-Erel et al. 2011; Erol-Dayi et al. 2011; Forgo et al. 2012).

In this study, the compounds from the chloroform extract of *C. kilaea* were isolated and structurally elucidated by spectroscopic methods. Also, these compounds were tested for cytotoxic activity against one normal (L-929; mouse fibroblast cell line) and three human cancer cell lines (Hela; cervix adenocarcinoma, MCF-7; breast adenocarcinoma, PC-3; prostate adenocarcinoma) using the MTT assay.

## Materials and methods

### Plant material

Aerial parts of plant were collected in the flowering periods from the Catalca region of Istanbul on July, 2009 and identified by Dr Gizem Bulut, a botanist of the Faculty of Pharmacy, University of Marmara. Voucher specimens were deposited in the Herbarium of the Faculty of Pharmacy, Marmara University (MARE No: 11712).

### Extraction

Extracts and sub-fractions of *C. kilaea* were obtained in our previous study (Sen et al. 2015). Briefly, dried aerial parts of *C. kilaea* were macerated separately in *n*-heptane, chloroform and methanol, with yield of 2.07, 4.01 and 8.40%, respectively. The chloroform extract (20 g), which showed anti-proliferative activity, was subjected to a silica gel column, and eluted by gradient elution (hexane-CHCI_3_-CH_3_OH) to afford 20 fractions. Fractions were combined according to their TLC behaviour to yield CKCSI (F4-10), CKCSII (F11-14) and CKCSIII (F15-20). All extracts were filtered, concentrated and dried under vacuum on a rotary evaporator at 40 °C and stored in a refrigerator for further analysis.

### Cell growth inhibition assay

Anti-proliferative activity of compounds (0.5–50 μg/mL) against three human cancer cell lines were carried out by MTT test as specified in our previous study (Sen et al. 2015). Compounds were tested for their cytotoxic activities. Cell viability and cytotoxic activity profile of the compounds were analyzed using the MTT assay (Mosman 1983; Woerdenbag et al. 1986; Beekman et al. 1997). MTT is cleaved to formazan by the ‘succinate-tetrazolium reductase’ system (EC 1.3.99.1) which belongs to the mitochondrial respiratory chain and is active only in viable cells. Different cell lines were used for the determination of cytotoxic activity [HeLa; (ATCC^®^, CCL- 2™), MCF-7 (ATCC^®^, HTB-22™), PC-3; (ATCC^®^, CRL-1435™)]. The cells were cultured with DMEM (Dulbecco’s Modified Eagle’s Medium) supplemented with 10% FBS (Foetal bovine serum), 1% l-glutamine and antibiotic solutions (penicillin–streptomycin–amphotericin).

*In vitro* cytotoxicity test was done by the method of modified Woerdenbag et al. (1986). The MTT metabolic assay was carried out with cells seeded at a density of 1 × 10^4^ cells/well in 96-well flat-bottom cell culture plates with 100 μL of opti-MEM and 24–48 h incubation at 37 °C, 5% CO_2_. The following day, media was aspirated and the compounds were dissolved in DMSO and diluted with medium before they were added to the cell cultures at different concentrations. Cells were incubated for 48 h at 37 °C, 5% CO_2_. After the incubation period, 10 μL of the MTT labelling reagent [final concentration 0.5 μg/mL (Cell proliferation kit MTT, Roche, Germany)] was added to each well. The cultures were incubated for 4–12 h in a humidified atmosphere (e.g., 37 °C, 5% CO_2_) and 100 μL of the solubilization buffer was added into each well. The plate was allowed to stand overnight in the incubator in a humidified atmosphere (e.g., 37 °C, 5% CO_2_), the formazan precipitates were solubilized. Absorbance of the formazan product was measured spectrophotometrically at 550 and 690 nm.

### Isolation of compounds from active CKCSII

**Figure 1.**
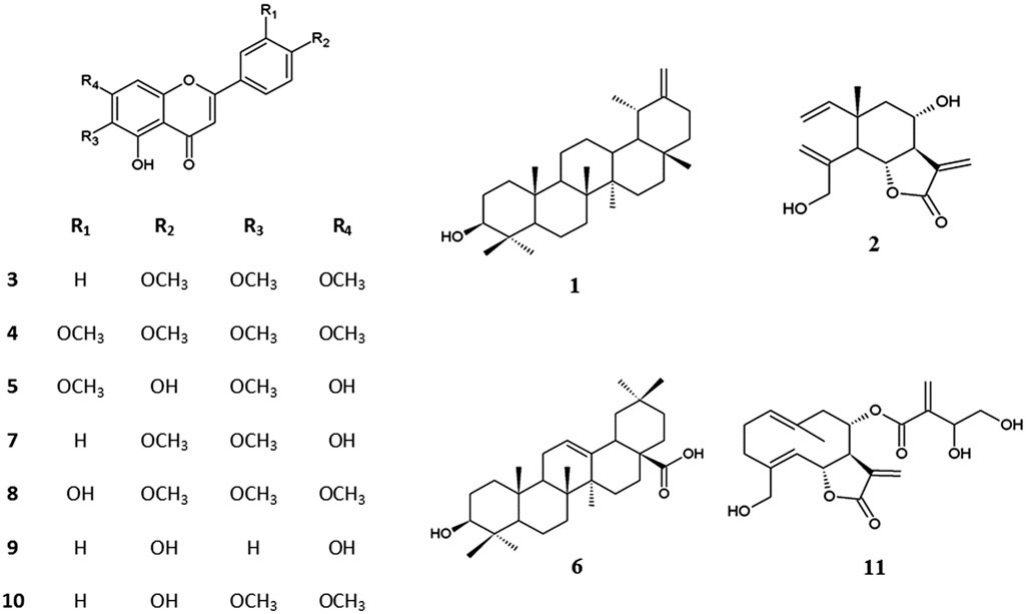
Chemical structures of 1–11 isolated from *C. kilaea*.

CKCSII exhibited the best anti-proliferative activity against human tumour cell lines according to our previous study (Sen et al. 2015). Therefore, we attempted to isolate compounds responsible for activity of CKCSII in this study. Fraction of CKCSII (18 g) was applied to vacuum liquid chromatography on normal-phase silica gel material (0.063–0.200 mm), using petroleum ether:diethyl ether:EtOAc:EtOH mixtures with increasing polarity to yield nine main fractions. Subfraction (CKCSII/5-8) (0.7135 g) was subjected to Sephadex LH-20 column, three times, eluted with CHCI_3_:MeOH (1:1). The combined subfractions were fractionated by preparative TLC, using petroleum ether:CHCI_3_: EtOAc (2:6:1) to yield taraxasterol (199.1 mg) (1). Sub-fraction (CKCSII/10-11) (0.636 g) was repeatedly chromatographed on a Sephadex LH-20 column, eluted with CHCI_3_:MeOH (2:1) and then combined sub-fractions was rechromatographed by preparative TLC with toluene/acetone (4:1) to give pure dehydromelitensin (9.3 mg) (2), salvigenin (49.7 mg) (3), 3′-*O*-methyleupatorin (35.7 mg) (4), and jaceosidin (9.1 mg) (5), with hexane:ethyl acetate (3:1) to yield oleanolic acid (9.3 mg) (6). Sub-fraction (CKCSII/12-14) (1.2972 g) was loaded to vacuum liquid chromatography on silica gel (0.063–0.2 mm) using petroleum ether with increasing amounts of EtOAc to afford 22 fractions. It was purified pectolinarigenin(12.5 mg) (7) from sub-fractions of (CKCSII/12-14/7), (CKCSII/12-14/8) and (CKCSII/12-14/9); eupatorin (38.6 mg) (8) from sub-fractions of (CKCSII/12-14/8) and (CKCSII/12-14/9); apigenin (4.2 mg) (9) from sub-fraction of (CKCSII/12-14/9); cirsimaritin (10.2 mg) (10) from sub-fraction of (CKCSII/12-14/9) by preparative TLC (CHCI_3_:petroleum ether:EtOAc =2.5:2:1). Sub-fraction (CKCSII/19) (0.6366 g) was submitted to Sephadex LH-20 column, two times, eluted with CHCI_3_:MeOH (1:1).The combined sub-fractions based on TLC profiles were purified by preparative TLC to obtain cnicin (37.3 mg) (11). For this, the plates were run three times in CHCI_3_:petroleum ether:EtOAc (2:2:1), subsequently twice in CHCI_3_:petroleum ether:EtOH (5:4:1.5) (Figure 1).

## Results

In this study, two sesquiterpene lactones, cnicin and dehydromelitensin, two triterpenes, oleanolic acid and taraxasterol, seven flavonoids, 3′-*O*-methyl eupatorin, apigenin, cirsimaritin, eupatorin, jaceosidin, pectolinarigenin and salvigenin, were isolated from active CKCSII sub-fraction of chloroform extract of aerial parts of *C. kilaea*. All isolated compounds were analyzed by spectroscopic methods (UV, ^1^H, ^13^C NMR-APT) and their data were compared with those reported in the literature. The compounds were identified as follows:

Taraxasterol: C_30_H_50_O: White amorphous powder (Khalilov et al. 2003; Yekta & Alavi 2008; Mouffok et al. 2012; Shakeri & Ahmadian 2014). ^1^H NMR (500 MHz, CDCI_3_, *δ*, ppm, J/Hz): 0.79 (3H, s, H-24), 0.87(3H, s, H-25), 0.88 (3H, s, H-28), 0.95 (3H, s,H-27), 0.99 (3H, s, H-23), 1.04 (3H, s, H-26), 1.04 (3H, d, *J* = 6.8 Hz, H-29), 3.23 (1H, dd, *J* = 11.4; 4.9 Hz, H-3), 4.62 (1H, *t*, *J* = 2.1 Hz, H-30a), 4.64 (1H, *t*, *J* = 2.1 Hz, H-30b). ^13^C NMR (125 MHz, CDCI_3_, *δ*, ppm): 38.8 (C-1), 27.4 (C-2), 79.1 (C-3), 38.8 (C-4), 55.4 (C-5), 18.3 (C-6), 34.1 (C-7), 40.9 (C-8), 50.5 (C-9), 37.1 (C-10), 21.5 (C-11), 26.2 (C-12), 39.2 (C-13), 42 (C-14), 26.7 (C-15), 38.3 (C-16), 34.5 (C-17), 48.6 (C-18), 39.4 (C-19), 154.7 (C-20), 25.6 (C-21), 38.9 (C-22), 28.0 (C-23), 15.4 (C-24), 16.3 (C-25), 15.9 (C-26), 14.8 (C-27), 19.5 (C-28), 25.5 (C-29), 107.2 (C-30).

Dehydromelitensin: Colourless oil: C_15_H_20_O (Sönmez et al. 1995; Salan & Oksuz 1999; Machado et al. 2012).^1^H NMR (500 MHz, CDCI_3_, *δ*, ppm, J/Hz):5.80 (H-1, dd, J: 17.4; 10.7 Hz, 1H), 5.06 (H-2a, d, 10.7 Hz, 1H), 5.00 (H-2b, d, *J* = 17,4 Hz, 1H), 5.42 (H-3a, s, 1H), 4.96 (H-3b, s, 1H), 2.54 (H-5, d, *J* = 11.8 Hz, 1H), 4.17 (H-6, t, *J* = 11.4 Hz, 1H), 2.67 (H-7, tt, *J* = 10.8; 2.9 Hz, 1H), 4.12 (H-8, m, overlapped, 1H), 1.88 (H-9a, dd, *J* = 13.1; 4.1 Hz, 1H), 1.67 (H-9b, m, overlapped, 1H), 6.19 (H-13a, d, *J* = 3.1 Hz, 1H), 6.02 (H-13b, d, *J* = 3.1 Hz, 1H), 1.11 (H-14, s, 3H), 4.10 (H-15a, d, *J* = 13.7 Hz, 1H), 4.01 (H-15b, d, *J* = 13.8 Hz, 1H). ^13^C NMR (125 MHz, CDCI_3_, *δ*, ppm): 146.20 (C-1), 112.70 (C-2), 114.88 (C-3), 143.95 (C-4), 50.60 (C-5), 78.88 (C-6), 55.04 (C-7), 67.46 (C-8), 49.75 (C-9), 41.94 (C-10), 137.47 (C-11), 169.97 (C-12), 120.53 (C-13), 18.88 (C-14), 67.30 (C-15).

Salvigenin: C_18_H_16_O_6_: Yellow amorphous powder. (Mabry et al. 1970; Salan et al. 2001). UV (MeOH, *λ*_max_, nm): 277, 329; +NaOMe: 278, 328; +AlCl_3_: 263sh, 301, 359; +AlCl_3_/HCl: 262sh, 300, 351; +NaOAc: 280, 328; +NaOAc/H_3_BO_3_: 277, 332. ^1^H NMR (500 MHz, CDCI_3_, *δ*, ppm, J/Hz): 6.57 (s, 1H, H-3), 6.61 (s, 1H, H-8), 7.86 (m, 1H, H-2′), 7.05 (m, 1H, H-3′), 7.03 (m, 1H, H-5′), 7.88 (m, 1H, H-6′), 3.92 (s, 3H, OCH_3_), 3.95 (s, 3H, OCH_3_), 3.99 (s, 3H, OCH_3_), 12.80 (s, 1H, 5-OH).

3′-*O*-Methyl eupatorin: C_19_H_18_O_7_: Yellow amorphous powder (Mabry et al. 1970; Salan et al. 2001). UV (MeOH, *λ*_max_, nm): 242, 276, 339; +NaOMe: 241, 277, 338; +AlCl_3_: 261, 288, 371; +AlCl_3_/HCl: 258, 291, 361; +NaOAc: 277, 338; +NaOAc/H_3_BO_3_: 276, 341. ^1^H NMR (500 MHz, CDCI_3_, *δ*, ppm, J/Hz): 6.57 (s, 1H, H-3), 6.61 (s, 1H, H-8), 7.35 (d, *J* = 2.1 Hz, 1H, H-2′), 7.00 (d, *J* = 8.5 Hz, 1H, H-5′), 7.54 (dd, *J* = 8.5; 2.1 Hz, 1H, H-6′), 3.95 (s, 3H, OCH_3_), 3.99 (s, 3H, OCH_3_), 4.00 (s, 3H, OCH_3_), 4.01 (s, 3H, OCH_3_), 12.78 (s, 1H, 5-OH).

Oleanolic acid: C_30_H_48_O_3_: White amorphous powder (Martins et al. 2013; Irungu et al. 2014). ^1^H NMR (500 MHz, CDCI_3_, *δ*, ppm, J/Hz): 3.14 (dd, *J* = 11; 5.1 Hz, 1H, H-3), 5,21 (*t*, *J*= 3.6 Hz, 1H, H-12), 2.76 (dd, *J* = 14; 3.6 Hz, 1H, H-18), 0.91 (s, 3H, H-23), 0.71 (s, 6H, H-24, H-26), 0.84 (s, 3H, H-25), 1.07 (s, 3H, H-27), 0.83 (s, 3H, H-29), 0.86 (s, 3H, H-30). ^13^C NMR (125 MHz, CDCI_3_, *δ*, ppm): 38.43 (C-1), 27.19 (C-2), 79.05 (C-3), 38.77 (C-4), 55.24 (C-5), 18.32 (C-6), 32.64 (C-7), 39.29 (C-8), 47.65 (C-9), 37.09 (C-10), 22.95 (C-11), 122.63 (C-12), 143.61 (C-13), 41.64 (C-14), 27.70 (C-15), 23.42 (C-16), 46.54 (C-17), 41.02 (C-18), 45.90 (C-19), 30.69 (C-20), 33.82 (C-21), 32.46 (C-22), 28.12 (C-23), 15.56 (C-24), 15.34 (C-25), 17.07 (C-26), 25.94 (C-27), 183.14 (C-28), 33.08 (C-29), 23.59 (C-30).

Jaceosidin: C_17_H_14_O_7_: Yellow amorphous powder (Mabry et al. 1970; Salan et al. 2001). UV (MeOH, λ_max_, nm): 274, 343; +NaOMe: 257, 265, 337sh, 406↑; +AlCl_3_: 262, 280, 376; +AlCl_3_/HCl: 259, 287, 364; +NaOAc: 275, 320sh, 394; + NaOAc/H_3_BO_3_: 275, 348. ^1^H NMR (500 MHz, CD_3_COCD_3_, *δ*, ppm, J/Hz): 6.66 (s, 1H, H-3), 6.73 (s, 1H, H-8), 7.66 (d, *J* = 2 Hz, 1H, H-2′), 7.03 (d, *J* = 8.4 Hz, 1H, H-5′), 7.63 (dd, *J* = 8.4; 2.1 Hz, 1H, H-6′), 3.89 (s, 3H, OCH_3_), 4.01 (s, 3H, OCH_3_), 13.27 (s, 1H, 5-OH).

Pectolinarigenin: C_17_H_14_O_6_: Yellow amorphous powder (Mabry et al. 1970; Salan et al. 2001; Chacón-Morales et al. 2013). UV (MeOH, *λ*_max_, nm): 276, 331; +NaOMe: 276, 369↓;  +AlCl_3_: 302, 357; +AlCl_3_/HCl: 301,351; +NaOAc: 275, 299sh, 368; +NaOAc/H_3_BO_3_: 277, 337. ^1^H NMR (500 MHz, CDCI_3_, *δ*, ppm, J/Hz): 6.44 (2H, H-3, H-8), 7.74 (d, *J* = 8.9 Hz, 2H, H-2′, H-6′), 6.90 (d, *J* = 8.9 Hz, 2H, H-3′, H-5′), 3.77 (s, OCH_3_), 3.81 (s, OCH_3_). ^13^C NMR (125 MHz, CDCI_3_, *δ*, ppm): 164.2 (C-2), 103.8 (C-3), 183.0 (C-4), 153.1 (C-5), 130.3 (C-6), 155.0 (C-7), 93.3 (C-8), 152.1 (C-9), 105.8 (C-10), 123.6 (C-1′), 128.1 (C-2′, C-6′), 114.5 (C-3′, C-5′), 162.6 (C-4′), 60.9 (4′-OCH_3_), 55.5 (6′-OCH_3_).

Eupatorin: C_18_H_16_O_7_: Yellow amorphous powder (Mabry et al. 1970; Oganesyan 2007; Yam et al. 2010). UV (MeOH, *λ*_max_, nm): 275, 340; +NaOMe: 276, 313sh, 371↓; +AlCl_3_: 260, 283, 369; +AlCl_3_/HCl: 257, 288, 360; +NaOAc: 276, 313sh, 370; +NaOAc/H_3_BO_3_: 277, 340. ^1^H NMR (500 MHz, CDCI_3_, *δ*, ppm, J/Hz): 6.63 (s, 1H, H-3), 6.61 (s, 1H, H-8), 7.36 (d, *J* = 2 Hz, 1H, H-2′), 7.00 (d, *J* = 8.5 Hz, 1H, H-5′), 7.54 (dd, *J* = 8.5; 2 Hz, 1H, H-6′), 3.99 (s, 3H, OCH_3_), 4.00 (s, 3H, OCH_3_), 4.06 (s, 3H, OCH_3_), 13.09 (s, 1H, 5-OH).

Apigenin: C_15_H_10_O_5_: Yellow amorphous powder (Mabry et al. 1970; Ersöz et al. 2002). UV (MeOH, *λ*_max_, nm): 268, 334; +NaOMe: 275, 326sh, 392↑; +AlCl_3_: 275, 301sh, 349, 381sh; +AlCl_3_/HCl: 277, 298sh, 344, 381sh; +NaOAc: 274, 307sh, 385; +NaOAc/H_3_BO_3_: 269, 340. ^1^H NMR (500 MHz, CD_3_COCD_3_, *δ*, ppm, J/Hz): 6.51 (s, 1H, H-3), 6.13 (d, *J* = 2.1 Hz, 1H, H-6), 6.43 (d, *J* = 2.1 Hz, 1H, H-8), 7.83 (m, 1H, H-2′), 6.92 (m, 1H, H-3′), 6.90 (m, 1H, H-5′), 7.81 (m, 1H, H-6′), 12.90 (s, 1H, 5-OH).

Cnicin: C_20_H_26_O_7_: White amorphous powder (Salan & Oksuz 1999; Csapi et al. 2010). ^1^H NMR (500 MHz, CDCI_3 _+ drops of CD_3_OD, *δ*, ppm, J/Hz): 5.10 (m, overlapped, 1H, H-1), 2.15–2.33 (m, H-2a), 2.15–2.33 (m, H-2b), 2 (m, overlapped, 1H, H-3a), 2.48–2.65 (m, H-3b), 4.87 (d, *J* = 10 Hz, 1H, H-5), 5.18 (*t*, *J* = 8.8 Hz, 1H, H-6), 3.17 (m, overlapped, 1H, H-7), 5.03 (m, overlapped, 1H, H-8), 2.48–2.65 (m, H-9a), 2.48–2.65 (m, H-9b), 6.23 (d, *J* = 3.2 Hz, 1H, H-13a), 5.81 (d, *J* = 2.6 Hz, 1H, H-13b), 1.52 (s, 3H, H-14), 4.25 (d, *J* = 13.7 Hz, 1H, H-15a), 4.02 (d, *J* = 13.8 Hz, 1H, H-15b), 4.53 (dd, *J* = 3.2; 6.4 Hz, 1H, H-3′), 3.75 (dd, *J* = 3.4; 11.3 Hz, 1H, H-4′a), 3.48 (dd, *J* = 6.7; 11.3 Hz, 1H, H-4′b), 6.39 (s, 1H, H-5′a), 6.11 (s, 1H, H-5′b). ^13^C NMR (100 MHz, CD_3_OD, δ, ppm): 130.90 (C-1), 26.92 (C-2), 35.20 (C-3), 145.56 (C-4), 129.78 (C-5), 78.75 (C-6), 54.06 (C-7), 74.53 (C-8), 49.05 (C-9, overlapped with solvent peak), 133.23 (C-10), 137.43 (C-11), 172.21 (C-12), 125.38 (C-13), 17.09 (C-14), 60.83 (C-15), 166.61 (C-1′), 142.29 (C-2′), 71.97 (C-3′), 66.67 (C-4′), 127.39 (C-5′).

Cirsimaritin: C_17_H_14_O_6_: Yellow amorphous powder (Mabry et al. 1970; Akkal et al. 2003; Bicha et al. 2011; Alwahsh et al. 2015). UV (MeOH, *λ*_max_, nm): 276, 333; +NaOMe:276, 387↑; + AlCl_3_: 285, 299sh, 362; +AlCl_3_/HCl: 287, 299sh, 355; +NaOAc: 273, 335sh, 387; +NaOAc/H_3_BO_3_: 275, 336. ^1^H NMR (500 MHz, CD_3_OD, *δ*, ppm, J/Hz): 6.57 (s, 1H, H-3), 6.73 (s, 1H, H-8), 7.80 (d, *J* = 8.5 Hz, 2H, H-2′, H-6′), 6.84 (d, *J* = 8.6 Hz, 2H, H-3′, H-5′), 3.74 (s, 3H, OCH_3_), 3.88 (s, 3H, OCH_3_).

As shown in Table 1, none of the isolated compounds displayed cytotoxic activity against L-929 and Hela cell lines (IC_50 _>_ _50 μg/mL). Cnicin exerted the remarkable anti-proliferative activity against MCF-7 cells with an IC_50_ value of 3.25 μg/mL while dehydromelitensin and pectolinarigenin showed moderate and weak activity, with IC_50_ values of 28.95 and 35.53 μg/mL, respectively. Cirsimaritin exhibited a strong anti-tumour activity against PC-3 cells with an IC_50_ value of 4.30 μg/mL, when dehydromelitensin demonstrated moderate activity, with an IC_50_ value of 25.00 μg/mL. Apigenin, cnicin, eupatorin and oleanolic acid also showed weak activity against PC-3 cells with IC_50_ values of 44.13, 31.53, 35.69 and 45.38 μg/mL, respectively (Table 1).

**Table 1.** Cytotoxic activity with normal and cancer cell lines of compounds isolated from *C. kilaea* (IC_50_, μg/mL).

	Cell lines			
Compounds	L-929	Hela	MCF-7	PC-3
Taraxasterol	-^a^	–	–	–
Pectolinarigenin	–	–	35.53 ± 2.70	–
Dehydromelitensin	–	–	28.95 ± 0.95	25.00 ± 0.44
3′-*O*-Methyl eupatorin	–	–	–	–
Salvigenin	–	–	–	–
Cnicin	–	–	**3.25 ± 0.03**	31.53 ± 0.71
Apigenin	–	–	–	44.13 ± 0.33
Eupatorin	–	–	–	35.69 ± 0.30
Oleanolic acid	–	–	–	45.38 ± 0.42
Jaceosidin	–	–	–	–
Cirsimaritin	–	–	–	**4.30 ± 1.55**

a IC_50 _>_ _50 μg/mL, inactive.

Results are expressed as the mean ± SD.

Bold values are significantly different from other values (p < 0.05).

## Discussion

Cancer is a condition of the cells dividing uncontrollably and might spread over to other tissues in contrast with normal cells dividing in a controlled way (Semary & Fouda 2015).

We previously reported anti-proliferative activity of heptane (H), chloroform (C) (its sub-fractions) and methanol extracts endemic *C. kilaea* against three human cancer cell lines (Hela; cervix adenocarcinoma, MCF-7; breast adenocarcinoma, PC-3; prostate adenocarcinoma) using MTT assay and C exhibited the greatest anti-proliferative activity against Hela and MCF-7 cells while C and M showed the highest activity against PC-3 cell. Three main fractions of C (CKCSI, CKCSII, CKCSIII) showing the most activity were tested and CKCSII demonstrated the highest activity against Hela and MCF-7 cells (Sen et al. 2015).

The aim of this study was to isolate anti-proliferative compounds from chloroform extract of *C. kilaea*. Total 11 compounds from the active extract of the plant consisting of seven flavonoids (3′-*O*-methyl eupatorin, apigenin, cirsimaritin, eupatorin, jaseosidin, pectolinarigenin, salvigenin), two sesquiterpene lactones (cnicin, dehydromelitensine) and two triterpene (oleanolic acid, taraxasterol) are isolated and the structures of compounds are elucidated using spectroscopic methods. Cirsimaritin, cnicin, dehydromelitensine, eupatorin, oleanolic acid and taraxasterol were isolated for the first time from *C. kilaea* in the present study.

Against MCF-7 cell line, cnicin has demonstrated quite strong cytotoxic activity with the IC_50_ value of 3.25 μg/mL. In previous studies, it was reported that cnicin compound was active on MCF-7 cell lines (4.2 μM or 1.59 μg/mL; another study 16.84 μM or 6.37 μg/mL) and its activity has been verified by this study (Bruno et al. 2005; Csapi et al. 2010). Also, Erel et al. (2011) showed that cnicin has cytotoxic effect towards different cancer cell lines; human malignant melanoma (SK-MEL) and human ductal carcinoma (BT-549) cells. Sesquiterpene lactones are known to be good anti-cancer agents. It is encountered in the literature that clinical trials are existed on a few substances (tapsigargin, artemisinin, parthenolide) (Ghantous et al. 2010). In previous studies, the fact that α-methylene-γ-lactone moiety played an important role in cytotoxic and pro-apoptotic effects of sesquiterpene lactons has been found. It has been claimed that α-methylene-γ-lactone moiety performed these effects by reacting with biological nucleophiles like thiol residues by means of Michael-type addition (Ghantous et al. 2010; Chicca et al. 2011). In our study, the effect of sesquiterpene lactone (cinicin) against cancerous cell lines could be considered to result from α-methylene-γ-lactone rings in their structures.

Cirsimaritin indicated quite strong anti-cancer activity with the IC_50_ value of 4.30 μg/mL against PC-3 cell line. The significant effect of cirsimaritin against the PC-3 has been revealed for the first time by this study. In a previous review study, it was reported that catechin, epicatechin, quercetin, kaempferol, luteolin, genistein, apigenin, myricetin and silymarin had cytotoxic activity against human prostate cancer cell line (Ren et al. 2003). Furthermore, it was suggested that the number and position of hydroxyl and methoxyl groups (especially, the position, the number and substitution of hydroxyl groups in the B ring) C4 carbonyl group, C2–C3 double bond, are important structure elements for anti-proliferative activity of flavonoids (Ramanouskaya et al. 2009; Sghaier et al. 2011). One or more of these substituent groups could have contributed to cytotoxic effect of cirsimaritin against PC-3 cell line.

The American National Cancer Institute (NCI) claims that compounds having an IC_50_ value that is equal to or less than 4 μg/mL could be potential anti-cancer drugs on cancerous cell lines (Alejandre-García et al. 2015). Therefore, it is seen that cnicin and cirsimaritin could be potential anti-cancer drug candidates against breast and prostate cancer, respectively.

## Conclusions

The active compounds of chloroform extract of *C. kilaea* showing activity have been found for the first time in this study. Dehydromelitensine, oleanolic acid, eupatorin, cnicin, cirsimaritin, taraxasterol have been isolated for the first time. Also, the results show that cnicin and cirsimaritin, isolated from chloroform extract of *C. kilaea*, are promising candidates to be anti-cancer drugs.
